# Traits of Orthorexia Nervosa and the Determinants of These Behaviors in Elite Athletes

**DOI:** 10.3390/nu12092683

**Published:** 2020-09-02

**Authors:** Olga Surała, Jadwiga Malczewska-Lenczowska, Dorota Sadowska, Izabela Grabowska, Agata Białecka-Dębek

**Affiliations:** 1 Institute of Sport—National Research Institute, 01-982 Warsaw, Poland; olga.surala@insp.waw.pl (O.S.); jadwiga.malczewska@insp.waw.pl (J.M.-L.); dorota.sadowska@insp.waw.pl (D.S.); 2 Faculty of Physical Education and Health in Biała Podlaska, Józef Piłsudski University of Physical Education in Warsaw, 21-500 Biała Podlaska, Poland; izabela.grabowska@awf-bp.edu.pl; 3 Department of Human Nutrition, Institute of Human Nutrition Sciences, Warsaw University of Life Sciences, 02-787 Warsaw, Poland

**Keywords:** athletes, ORTO-15, DXA—body composition, sex, type of sports

## Abstract

Background: The purpose of this study was to evaluate the traits of orthorexia nervosa (ON) and its relation to body composition and anthropometric indices among elite athletes. Methods: We studied 273 (125 female, 148 male) competitive athletes. ON was assessed with the self-administered ORTO-15 questionnaire. Total body composition was determined using dual-energy X-ray absorptiometry (DXA). Results: The ORTO-15 score was independent of sex, type of effort or age. The ORTO-15 score was related to: total body mass (TBM) (*p* = 0.037; *R* = 0.170), body mass index (BMI) (*p* = 0.022; *R* = 0.187), bone mineral content (BMC) (*p* = 0.035; *R* = 0.172), lean soft tissue (*p* = 0.026; *R* = 0.182) and visceral adipose tissue (VAT) (*p* = 0.007; *R* = 0.255) in the male; BMI (*p* = 0.045; *R* = 0.412) and BMC (*p* = 0.012; *R* = 0.506) in weight-dependent male athletes. There was no relationship between ORTO-15 score and body composition in the total study group and subgroup of female athletes. In female athletes, ON tendencies were related to the weekly training time (*p* = 0.039), but in sprint and high-intensity intermittent efforts subgroup exclusively. Conclusions: Factors related to ON tendencies concerned exclusively BMI and body composition in male, and the weekly training time in female athletes. The results from this study demonstrate that a significant percentage of athletes, irrespective of sex, age, type of sport or hours of training per week, may manifest traits of orthorexia nervosa.

## 1. Introduction

Orthorexia nervosa (ON) is a behavioral pattern described as a pathological fixation on healthy nutrition. Orthorexic individuals are typically concerned more about the quality of food in their diet than the quantity, which leads to an extremely restrictive diet, and consequently might contribute to nutritional deficiencies, malnourishment, medical complications and reduced quality of life [1]. Although there have been a few attempts to introduce diagnostic criteria of ON, none have been officially accepted so far. Therefore, currently, ON is not recognized as an eating disorder in the Diagnostic and Statistical Manual of Mental Disorders (DSM-5) or the International Statistical Classification of Diseases and Related Health Problems (ICD-10) [2,3].

Some studies suggest that there is a link between ON and exercise habits [4,5,6,7]. Involvement in sport, particularly at the elite level, creates many nutritional challenges. Accordingly, it is common for athletes to maintain control over their diet in order to optimize training adaptations, enhance their performance and improve recovery or to achieve desired body weight and/or body composition [8]. Some athletes approach their dietary habits in such a restrictive and rigid way that it can lead to eating disorders. Indeed, eating disorders are reported to be more common in athletes compared to the general population [9,10]. Studies show that, excluding athletes with muscle dysmorphia [11], the prevalence of eating disorders is higher among female (6–45%) then male athletes (0–19%), and it is more frequent in certain types of sport (e.g., aesthetic sports, weight-dependent sports and endurance sports) [9,12].

Little is known about the prevalence of ON in sport. The results of only few studies indicate that ON occurs more often in athletes than in nonathletes [13,14]. Even less is known about the relationship between ON and body composition in athletes. To the best of our knowledge, only one study, by Agopyan et al. (2019) [15], has evaluated the association between body composition and ON; however, participants of the study were female students of nutrition and dietetics, not athletes. In light of these limitations, the purpose of this study was to evaluate the traits of orthorexia nervosa among athletes from a range of sports. Moreover, we aimed to investigate the relationship between orthorexic behaviors expressed as ORTO-15 score and body composition, anthropometric indices and other factors such as age, sex, type of sport, physical loads and training experience in elite athletes.

We expected that risk of ON would be related to some parameters of body composition (i.e., percent body fat and lean soft tissue mass) as well as training experience. Our second hypothesis was that athletes participating in weight-dependent and endurance sports would present more orthorexic behaviors than other sports.

## 2. Materials and Methods

### 2.1. Participants

A cohort of 273 (125 female, 148 male) competitive athletes from nineteen different Olympic sports participated in this study. Subjects were mostly either members of senior or youth national teams. Based on the type of effort performed during competition and on the characteristics of the sports, athletes were stratified into four groups (Table 1): high-intensity intermittent efforts: athletics, badminton, football, indoor and beach volleyball (*n* = 59), endurance: athletics (middle and long distance runners), biathlon, cross country skiing, road, mountain bike and track cycling, modern pentathlon, Nordic combined, swimming, triathlon (*n* = 132), sprint: canoe sprint, track cycling (*n* = 36) and weight-dependent sports (including aesthetic sports due to there being only two representatives of this type of sport): figure skating, judo, rowing lightweight, taekwondo (*n* = 46). Written informed consent was obtained from the subjects or their parents or legal guardian if the participant was under the age of consent. Inclusion criteria included age of 14.0–39.9 years, professional sport engagement of ≥2 years. Due to methodological limitations of dual-energy X-ray absorptiometry (DXA), the exclusion criterion was height >194 cm. The study was granted ethical approval (KEBN-20-53-0S) by the Ethics Committee of the Institute of Sport—National Research Institute, Poland, in agreement with the Helsinki Declaration.

### 2.2. Orthorexia Nervosa Measure

ON was assessed with the Polish adaptation of the self-administered ORTO-15 questionnaire. The Polish version was adapted from the original Italian ORTO-15 [16,17] and has satisfactory reliability (Cronbach’s Alpha 0.70–0.90) [17]. The ORTO-15 includes 15 questions with response options in the form of a four-level Likert scale. Answers to each question are scored from 1 to 4 points, with lower numbers corresponding to more traits of orthorexia. The total ORTO-15 score ranges from 15 to 60, with the value 40 [16,18], 35 [17,19] or 24 [20] being considered the cut-off point below which ON tendencies are indicated.

### 2.3. Body Composition Assessment

Whole-body composition measurements were performed using a Lunar Prodigy Pro DXA device (GE Healthcare, Madison, USA); whole-body scans were analyzed using GE Encore v16 software. Body composition assessments were performed under standardized scanning conditions. Participants were measured in the morning hours (7:30–9:30 a.m.) following an overnight fast, wearing only underwear, with all metal objects removed and bladder voided. Each day, right before the examination, the DXA machine was calibrated with a quality assurance calibration block. The following whole-body composition estimates were assessed: fat tissue (FAT, kg), percent body fat (FAT%), lean soft tissue mass (LST, kg), bone mineral content (BMC, g). Visceral adipose tissue mass (VAT, g) was additionally measured in athletes over 18 years of age.

### 2.4. Statistical Analyses

Collected data were processed using Statistica v.13. Continuous variables were reported as means (x) and standard deviations (SDs), whereas categorical variables were presented as absolute frequency (*n*) and percentage (%). Normality of the continuous variables was tested with the Shapiro-Wilk test. For most variables, the assumptions of normal distribution were not met; therefore, nonparametric tests were performed. Association between two continuous variables was assessed with the Mann-Whitney U test, whereas for multiple comparisons the Kruskal–Wallis one-way analysis of variance was used, and the Dunn test as a post hoc test. The correlations between normally distributed variables were analyzed with the Spearman’s rank-order test (R). The chi-square statistic (χ^2^ test) was used to compare differences in the frequencies of categorical variables. Test significance was set at an α level of *p* < 0.05. Due to statistically significant differences between female and male athletes in terms of body composition and anthropometric indices, the relationship between them and ORTO-15 score was assessed separately for each sex.

## 3. Results

Mean age of the participants was 20.9 ± 4.7 years (range: 14.1–38.8 years). They were involved in professional training for 10.2 ± 4.8 years (range: 2.0–24.0 years), and they spent on average 17.7 ± 6.0 h per week exercising (range: 4.5–40 h/week). The detailed characteristics of the study participants are displayed in Table 2.

The mean ORTO-15 score for the entire group was found to be 35.3 ± 3.5 (range: 25–47 pt.), and there were no differences between sexes according to mean ORTO-15 score (*p* = 0.506) or in the prevalence of the scores below the fixed cut-off value of 35 (*p* = 0.756) or 40 points (*p* = 0.168). Similarly, no effort-type related differences were observed for any of the ORTO-15 results, either in the entire population, or in the subgroups based on sex (Table 3). Moreover, no significant difference was observed between adults (≥18 years) and underage (<18 years) athletes in relation to ORTO-15 score (adults 35.3 ± 3.4 vs. underage 35.3 ± 3.6; *p* = 0.793), or according to prevalence of orthorexic tendencies stated on the basis of the cut-off value of 35 (adults 39.4% vs. underage 45.9%; *p* = 0.311) or 40 (adults 87.8% vs. underage 89.4%; *p* = 0.695). Additionally, none of the studied athletes scored below 25 points in ORTO-15 questionnaire.

No relationship was observed between ORTO-15 score and body composition indices in the total study group and subgroup of female athletes (Table 4). However, in the male subgroup there was a very weak positive linear correlation between ORTO-15 score and TBM (*p* = 0.037; *R* = 0.170), BMI (*p* = 0.022; *R* = 0.187), BMC (*p* = 0.035; *R* = 0.172), LST (*p* = 0.026; *R* = 0.182) and VAT (*p* = 0.007; *R* = 0.255).

Due to the small sample size of the female high-intensity intermittent efforts group (*n* = 20), for analysis of correlation, high-intensity intermittent efforts and sprinter athletes were classified as the other sports group. In the group of female athletes, regardless of the type of effort, there was no relationship between ORTO-15 score and anthropometric measurements or body composition. There was only a relationship between ORTO-15 score and training time (*p* = 0.039; *R* = −0.252) in the other sports (i.e., sprint and high-intensity intermittent efforts) subgroup (Table 5).

Due to the small sample size of the male sprint group (*n* = 13), for analysis of correlation, sprinter and high-intensity intermittent efforts athletes were classified as the other sports group. Taking into account the distribution into three effort-type groups, in male athletes ORTO-15 score was positively related to BMI (*p* = 0.045; *R* = 0.412) and BMC (*p* = 0.012; *R* = 0.506) only in weight-dependent sports (Table 6).

## 4. Discussion

This is the first study to analyze the relationship between body composition measured with DXA and risk of ON in elite athletes, and one of the very few to investigate the risk of ON in this subpopulation.

In the Polish population a score of <24 indicates a strong preoccupation with consuming healthy food [20]. However, none of the studied athletes scored below 25 points in the ORTO-15 questionnaire. Moreover, the results of our study indicate that depending on the adopted cut-off value of 35 or 40 points, 41.3% to 88.3% of participants, respectively, manifested ON tendencies. The prevalence of scores below either 35 or 40 points found in our group is higher than observed in previous studies concerning athletes. Segura-Garcia et al. (2012) [14], using a cut-off point of 35, reported that the frequency of occurrence of ON among Italian athletes was 28% in females and 30% in males, whereas Clifford and Blyth (2019) [13], who set a threshold of 40 points to indicate ON tendencies in student athletes, found that 76% of participants scored below 40. Moreover, Bert et al. (2019) reported that in Italian athletes, who practiced endurance physical activity for >150 min/week, a mean ORTO-15 score lower than 35 and 40 was observed in 21.5% and 72.8% of subjects, respectively [21]. The discrepancies in our findings and the results reported by other researchers [13,14,21] might result from different competitive levels of studied athletes, since some data indicate that the higher the level of athleticism, the greater the likelihood of disordered eating behaviors [22] and dietary restrictions [23]. In our study all participants were elite athletes competing at national and international levels, whereas subjects participating in previous studies were either both recreational and professional athletes [14], members of university teams [13] or athletes competing only in local level events [21]. Moreover, the higher frequency of ON in our study group might also be explained by the high mean weekly volume of training (17.6 ± 6.0 h/week), because according to recent findings, the ON symptom rate is higher in athletes who spend >10 h per week training [13] or participate in endurance activities for >150 min/week [21].

It should be emphasized that we found a relationship between the number of hours of training per week and ON tendencies solely in the other sports (i.e., sprint and high-intensity intermittent efforts) female subgroup. Our results corroborate earlier findings of Eriksson et al. (2008), who reported that ON behaviors increased with the increase of self-reported exercise frequency, but only in female fitness participants [24]. The relationship between time spent on exercise and the ON symptoms rate was also confirmed by other studies conducted in the student’s population [5,25], among athletes [13,21] and gym attendees [4,6].

A recently published systematic review on sex differences in the frequency and levels of ON behaviors in the general population shows that the prevalence of pathologically healthy eating is somewhat higher among women than men [26]. Nonetheless, we did not observe any association between ON tendencies and sex. Our observations are in line with the results of studies aiming to analyze the issue of ON in sport [13,14,21]. Similar to our results, the authors of those studies also did not observe any differences between male and female athletes in the severity of orthorexic tendencies. Moreover, our study did not reveal any relationship between ON and type of sport, which contradicts the literature on eating disorders in athletes [12,27,28,29,30]. According to the narrative review by Joy et al. (2016), athletes who participate in sports in which having a low body mass is perceived as advantageous for performance, as well as in weight class sports and aesthetic sports, are more likely to engage in pathological eating behaviors [12]. Nevertheless, our results are in agreement with a study by Clifford and Blyth (2019), who did not observe any significant differences in ORTO-15 score between students who competed in weight-dependent sports and other student athletes [13].

Our findings of a similar, high rate of ON tendencies among female and male athletes, regardless of age or type of sport, raise questions of whether, and to what extent, this is due to a pathological obsession with healthy eating, or if it instead stems from a high preoccupation of proper nutrition aiming for high physical performance. The latter explanation may be supported by the results of a study by Pelly et al. (2018), who investigated factors in food decision-making during the Melbourne 2006 and Delhi 2010 Commonwealth Games [31]. When making food choices, the athletes placed factors influencing their performance (e.g., nutrient composition) above the factors relating to the sensory value of food [31]. Furthermore, most athletes are aware that optimal nutrition is an essential and integral element of a training program [32], which might influence their eating habits, not necessarily increasing the pathological behaviors. Therefore, it might be supposed that the ORTO-15 questionnaire may not be accurate enough for the identification of orthorexia nervosa in athletes. Despite the fact that ORTO-15 is the most frequently used tool to assess ON, it has met with some criticism [33]. Some authors claim that, presumably, the ORTO-15 fails to distinguish between healthy dieting and pathologically healthful eating. Thus, it likely overestimates the prevalence of ON in the general population [34,35]. To dispel these doubts, future research is needed, with simultaneous assessment of diet and nutrition in the subpopulation of athletes.

In the present study there was a weak correlation between orthorexic tendencies and BMI in male, but not in female athletes. The effects of BMI on the risk of ON have been widely studied so far, but the results are inconsistent. There are a few studies which found that ON was related to BMI [36,37]. However, in most studies, the authors did not observe any correlation between ON and BMI classification [5,15,38,39,40]. So far only Agopyan et al. (2019) assessed the relationship between ON score and body composition measured with bioelectrical impedance analysis in female students of nutrition and dietetics [15]. No relationship was identified in the studied group. Similarly, in our study, despite the use of a more advanced method of body composition assessment (DXA), in the female athletes, regardless of the type of sport, no relationship was observed between ON score and body composition indices. However, in our findings, in the whole male subgroup as well as in one of the sport subgroup, orthorexic tendencies were related both to anthropometric and body composition indices. Higher TBM and LST in males with higher orthorexic tendencies explain the correlation between BMI and ORTO-15 in this subpopulation. This relationship may indicate that athletes who manifested lower ON tendencies could have less restrictive eating behaviors, which could translate into sufficient energy and macronutrient intake, which is essential for achieving or maintaining higher lean body mass [8,41]. Accordingly, this supposition could explain why adult males with less strict diets had higher VAT, and why athletes from weight-dependent sports had higher BMC [42,43]. However, in order to confirm these hypotheses, a thorough analysis of the diet and nutrition of subjects would be necessary, which was outside the scope of this study.

This study has some limitations. The main limitation of this study is the application of only one tool for assessment of traits of ON i.e., the ORTO-15 questionnaire. Utility of the ORTO-15 that was used in presented study has been widely discussed, and some authors claim that this tool measures only healthy eating, and not pathologically healthful eating [19,44]. However, despite many flaws, the ORTO-15 is still the most commonly used psychometric tool [45,46,47,48,49]. There are a few diagnostic tools that are used to assess orthorexia nervosa in general population (ex. The ORTO-15, The EHQ (Eating Habits Questionnaire), the DOS (Dusseldorf Orthorexia Scale), the BOS (Barcelona Orthorexia Scale), and the TOS (Teruel Orthorexia Scale)), but only the ORTO-15 has been validated to the Polish conditions. It is worth noting that currently there is not a reliable tool to objectively assess the phenomenon of ON in the subpopulation of athletes. Furthermore, the study group was at the same time a limitation and a strength of this study. On one hand, the study group consisted of elite athletes, in a wide age range, from various sports, making it a representative sample of this subpopulation. On the other hand, the heterogeneity of the studied group made it difficult to make conclusions about the relationship between ON, body composition and anthropometric indices. Further studies should be conducted on a larger sample of athletes and should take into account: (1) more detailed analysis of eating habits with the Food Frequency Questionnaire (FFQ-6) or Eating Habits Questionnaire (EHQ), or other tools assessing habitual diet; (2) detailed analysis of food attitudes or symptoms and concerns typical for eating disorders, for example by using the Eating Attitude Test 26 (EAT-26) or the Brief Eating Disorder in Athletes Questionnaire (BEDA-Q) if female athletes were studied; (3) a broader analysis of ON risk with tools such as ORTO-15 accompanied by the BOS, DOS, EHQ or TOS questionnaires.

## 5. Conclusions

The results from this study demonstrate that a significant percentage of athletes, irrespective of sex, age, type of sport or hours of training per week, may manifest traits of orthorexia nervosa. In contrast to our expectations, the ON’s risk was not related to percent body fat and lean soft tissue mass or training experience. Moreover, no effort-type related differences were observed for the risk of ON. Factors related to ON tendencies observed in male athletes, solely participants of weight-dependent sports, concerned BMI and BMC. Whereas in female athletes, ON tendencies were related to the weekly training time, but in sprint and high-intensity intermittent efforts subgroup exclusively. Given that elite athletes are a group with specific nutritional needs, it seems worthwhile to create tools dedicated strictly for sport, which would allow for a more precise diagnosis of ON symptoms. For this purpose, future analysis is needed in this area with a simultaneous assessment of athletes’ diet and eating habits.

## Figures and Tables

**Table 1:** Study population by type of effort and sport disciplines. Data presented as number (%).

Effort-Type Group	Sport	Total *n* (%)	Female *n* (%)	Male *n* (%)
High-intensity intermittent efforts (*n* = 59)	Athletics	2 (0.7)	1 (0.8)	1 (0.7)
	Badminton	16 (5.9)	6 (4.8)	10 (6.8)
	Football	10 (3.7)	0 (0)	10 (6.8)
	Volleyball: indoor	5 (1.8)	0 (0)	5 (3.4)
	beach	26 (9.5)	13 (10.4)	13 (8.8)
Endurance (*n* = 132)	Athletics	1 (0.4)	0 (0)	1 (0.7)
	Biathlon	28 (10.3)	13 (10.4)	15 (10.1)
	Cross country skiing	32 (11.7)	15 (12)	17 (11.5)
	Cycling: road	2 (0.7)	0 (0)	2 (1.4)
	mountain bike	10 (3.7)	2 (1.6)	8 (5.4)
	track	1 (0.4)	1 (0.8)	0 (0)
	Modern pentathlon	12 (4.4)	4 (3.2)	8 (5.4)
	Nordic combined	5 (1.8)	0 (0)	5 (3.4)
	Swimming	39 (14.3)	21 (16.8)	18 (12.2)
	Triathlon	2 (0.7)	2 (1.6)	0 (0)
Sprint (*n* = 36)	Canoe sprint	24 (8.8)	16 (12.8)	8 (5.4)
	Cycling: track	12 (4.4)	8 (6.4)	4 (2.7)
Weight-dependent sports (*n* = 46)	Figure skating	2 (0.7)	1 (0.8)	1 (0.7)
	Judo	28 (10.3)	15 (12)	13 (8.8)
	Rowing lightweight	7 (2.6)	3 (2.4)	4 (2.7)
	Taekwondo	9 (3.3)	4 (3.2)	5 (3.4)

**Table 2:** Characteristics of subjects and statistical differences by sex.

Variable	Total (*n* = 273)	Female (*n* = 125)	Male (*n* = 148)	*p* Value ^1^
	Mean ± SD	Mean ± SD	Mean ± SD	
Age (years)	20.9 ± 4.7	20.2 ± 4.5	21.5 ± 4.8	**0.009**
Training experience (years)	10.2 ± 4.8	9.4 ± 5.1	10.9 ± 4.3	**0.002**
Training (h/week)	17.6 ± 6.0	17.6 ± 5.7	17.7 ± 6.3	0.593
ORTO-15	35.3 ± 3.5	35.1 ± 3.1	35.4 ± 3.7	0.506
Height (cm)	176.5 ± 9.3	169.8 ± 7.2	182.1 ± 6.9	**<0.001**
TBM (kg)	69.9 ± 11.7	62.4 ± 8.1	76.3 ± 10.5	**<0.001**
BMI (kg/m^2^)	22.2 ± 2.5	21.5 ± 2.3	22.8 ± 2.5	**<0.001**
BMC (g)	2.9 ± 0.6	2.5 ± 0.3	3.3 ± 0.5	**<0.001**
FAT (kg)	11.6 ± 3.4	13.0 ± 3.6	10.4 ± 2.7	**<0.001**
FAT (%)	17 ± 5.2	20.8 ± 4.6	13.7 ± 3.2	**<0.001**
VAT (g)	59.2 ± 81.8	25.5 ± 50.9	82.6 ± 90.7	**<0.001**
LST (kg)	55.4 ± 11.1	46.8 ± 6.4	62.6 ± 9.0	**<0.001**

^1^ Mann-Whitney U test; Abbreviations: TBM—total body mass; BMI—body mass index; BMC—bone mineral content; FAT—fat tissue; FAT%—percent body fat; VAT—visceral adipose tissue; LST—lean soft tissue; statistically significant *p* values are in bold.

**Table 3:** Sample mean ORTO-15 score and prevalence of scores below fixed cut-off value of 35 or 40 points including sex and type of effort.

Total Sample	Total Sample (*n* = 273)	High-Intensity Intermittent Efforts (*n* = 59)	Endurance (*n* = 132)	Sprint (*n* = 36)	Weight-Dependent Sports (*n* = 46)	*p* Value
ORTO-15 (x ± SD)	35.3 ± 3.5	35.4 ± 3.8	35.2 ± 3.2	35.0 ± 3.5	36.6 ± 3.6	0.8627 ^a^
Cut-off [*n*(%)]:						
<35 pts	113 (41.4)	29 (49.2)	50 (37.9)	18 (50.0)	16 (34.7)	0.2546 ^b^
<40 pts	241 (88.3)	50 (84.7)	122 (92.4)	30 (83.3)	39 (84.8)	0.2310 ^b^
Female (*n* = 125)						
ORTO-15 (x ± SD)	35.1 ± 3.1	35.0 ± 3.9	35.3 ± 2.8	34.9 ± 3.7	35.2 ± 2.6	0.6129 ^a^
Cut-off [*n*(%)]:						
<35 pts	53 (42.4)	11 (55.0)	20 (34.5)	13 (54.2)	9 (39.1)	0.2357 ^b^
<40 pts	114 (91.2)	17 (85.0)	54 (93.1)	21 (87.5)	22 (95.7)	0.5325 ^b^
Male (*n* = 148)						
ORTO-15 (x ± SD)	35.4 ± 3.7	35.6 ± 3.8	35.0 ± 3.5	36.0 ± 3.3	36.1 ± 4.3	0.5217 ^a^
Cut-off [*n*(%)]:						
<35 pts	60 (40.5)	18 (46.2)	30 (40.5)	5 (41.7)	8 (30.4)	0.6844 ^b^
<40 pts	127 (86.0)	33 (84.6)	68 (91.9)	10 (76.9)	18 (75.0)	0.1059 ^b^

^a^ Kruskal-Wallis ANOVA; ^b^ χ^2^ test.

**Table 4:** Relationship between ORTO-15 score and anthropometric measurements, body composition, training experience and physical loads in athletes including sex.

Variables	ORTO-15		
	Total (*n* = 273)	Female (*n* = 125)	Male (*n* = 148)
Height (cm)	0.011	−0.151	0.043
TBM (kg)	0.078	−0.056	**0.170**
BMI (kg/m^2^)	0.103	−0.005	**0.187**
BMC (g)	0.085	−0.048	**0.172**
FAT (kg)	0.022	0.016	0.065
LST (kg)	0.074	−0.071	**0.182**
FAT%	−0.010	0.060	0.007
VAT (g)	0.121	−0.109	**0.255**
Training experience (years)	−0.002	−0.010	0.065
Training time (h/week)	0.000	−0.171	0.129

Spearman’s rank-order correlation; *p* < 0.05. Significant correlations values are in bold. Abbreviations: TBM—total body mass; BMI—body mass index; BMC—bone mineral content; FAT—fat tissue; LST—lean soft tissue; FAT%—percent body fat; VAT—visceral adipose tissue.

**Table 5:** Relationship between ORTO-15 score and anthropometric measurements, body composition and training time and experience in female athletes including type of effort.

Variables	Female Athletes		
	Endurance (*n* = 58)	Weight-Dependent Sports (*n* = 23)	Other Sports (*n* = 44)
Height (cm)	−0.176	−0.200	−0.137
TBM (kg)	−0.005	0.042	−0.063
BMI (kg/m^2^)	0.070	0.163	−0.009
BMC (g)	−0.131	0.071	0.045
FAT (kg)	0.100	0.184	−0.039
LST (kg)	−0.062	0.000	−0.032
FAT%	0.121	0.128	−0.031
VAT (g)	−0.266	−0.292	−0.064
Training experience (years)	0.071	−0.187	−0.135
Training time (h/week)	−0.120	−0.331	**−0.252**

Spearman’s rank-order correlation; *p* < 0.05 significant correlations values are in bold. Abbreviations: TBM—total body mass; BMI—body mass index; BMC—bone mineral content; FAT—fat tissue; LST—lean soft tissue; FAT%—fat tissue content; VAT—visceral adipose tissue.

**Table 6:** Relationship between ORTO-15 score and anthropometric measurements, body composition, training loads and training experience in male athletes including type of effort.

Variables	Male Athletes		
	Endurance (*n* = 74)	Weight-Dependent Sports (*n* = 24)	Other Sports (*n* = 52)
Height (cm)	0.106	0.023	−0.052
TBM (kg)	0.103	0.334	0.107
BMI (kg/m^2^)	0.001	**0.412**	0.179
BMC (g)	0.131	**0.506**	−0.051
FAT (kg)	0.125	0.375	−0.233
LST (kg)	0.104	0.282	0.184
FAT%	0.097	0.257	−0.250
VAT (g)	0.270	0.213	0.278
Training experience (years)	0.038	−0.121	0.057
Training time (h/week)	0.161	0.019	0.192

Spearman’s rank-order correlation; *p* < 0.05 significant correlations values are in bold. Abbreviations: TBM—total body mass; BMI—body mass index; BMC—bone mineral content; FAT—fat tissue; LST—lean soft tissue; FAT%—percent body fat; VAT—visceral adipose tissue.
